# Supplementary material for: Predicting adverse drug effects: A heterogeneous graph convolution network with a multi-layer perceptron approach
Source: PLoS One. 2022 Dec 14;17(12):e0266435. doi: 10.1371/journal.pone.0266435 (PMC9750037; doi:10.1371/journal.pone.0266435)
Supplement: Attachment — Submitted filename: Response_to_Reviewers_v2.pdf [file pone.0266435.s001.pdf]

Title: Predicting Adverse Drug Effects: A Heterogeneous Graph Convolution Network with a Multi-layer Perceptron Approach
Ref.: PONE-D-22-08273R1 by Chen, Shih, Chien and Tsai

Thank you very much for your letter of August 22. We have made the following changes according to the referees' suggestions in the above-mentioned revised manuscript, which we will resubmit to PLOS ONE for possible publication. First, we must apologize to Review #2 for not addressing two of his comments because of our carelessness in the first revision.

Reviewer #2:

1 *There are no references to similar studies of adverse drug effects prediction, and the related works must be included in the Introduction section.*
Response 1: Some articles related to adverse drug side effect prediction, and related works have been added in the References. More precisely, Refs [32-40, 43, 46] have been addressed in the Introduction of the first revision. In addition, Refs. [48], [54], and [67-69] have been addressed in p.5, p.7, and p.10, respectively.

2 *The whole dataset should be described clearly. For example, how many drugs, side effects, positive and negative links between drugs and side effects are in the dataset?*
Response 2: Please see the statements in the Datasets Section, p.4.

3 *The similarity score is the key to building the networks among drugs. Nevertheless, it is unclear how to apply the natural language processing method to calculate the similarity score between two drugs.*
Response 3: Similarity scores between the drug in the dataset is the cosine similarity derived from the word2vec package combined with a model pre-trained on PubMed, PMC, and Wikipedia texts by Pyysalo et al.'s work [47]. The word representations are derived from the large corpus of biomedical and general-domain texts, including PubMed abstracts (nearly 23 million abstracts) and PMC articles (about 700000 full texts), plus approximately four million English Wikipedia articles. We add the above in the Datasets section (page 4, lines 93–101).

4 *In Table 2, the authors compared the predictive performance by the different percentages of the training set. However, it is unknown the size of the testing set. It is better to use k-fold cross-validation to evaluate the performance appropriately.*
Response 4: We use k–fold cross–validation for $k = 6, 8, 10, 12$, and chose the independent testing dataset of 10%, 20% in the Experimental Results Section on lines 192–201 and revised Table 2 to make it clear.

5 *The predictive performance was compared with several algorithms (Table 4), but it should be compared with other related works.*
Response 5: We compared our method with the following machine learning models: collaborative filtering, logistic regression, k-nearest neighbor classifier, support vector machine, random forest, and gradient boosting classifier (see Table 7). We also describe on p. 11, lines 230–235

6 *The discussion section should be extended and elaborated.*
Response 6: The Conclusions section has been revised, where we indicated the strength of the GCNMLP in the first paragraph, and the weakness of the proposed network methods was addressed.

Reviewer #4:

1 *We strongly suggest the authors prepare an independent test set and report the performance of the proposed method based on it. Ten-fold cross-validation used in this study is for parameter optimization in machine learning. It could overestimate the ability of the proposed approach and result in an overfitting performance.*
Response 1: We added a 10% and 20% independent test set for evaluating the performance of the GCNMLP model. The results showed similar results as in Table 2 previously.

2 *It is necessary to compare the proposed method with other existing approaches (e.g., with a benchmark dataset) to prove the proposed one is*

better than the others. Did methods, such as NMF, NMFHD, PA, RA, etc., shown in Table 5 propose by others? If yes, please give appropriate citations or notes in Table 5.
Response 2: We compared the GCNMLP with previous work by Timilsina, et al. [43] for NMFHD with the benchmark dataset SIDER. The comparison methods have been suggested in [43].

3 *The abstract can be more detailed. Readers will appreciate the detailed information on the importance, innovation, and contributions of this research.*
Response 3: We have revised the abstract.

4 *We also suggest the authors release their machine learning models or source codes for further academic studies in the future*
Response 4: We have released the source codes in the GitHub as `https://github.com/yishingene/gcnmlp`.

5 *Please define the full name of NMFHD.*
Response 5: There was a typo in p. 5, line 107, namely, "NFMHD", which has been revised as "NMFHD". In addition, we gave a more detailed description of the heat equation. Thus the discussion concerning the NMFHD is more readable than that of the first revision.

Finally, the authors would like to thank reviewers for their crucial suggestions concerning our manuscript.
